# Application of AI in Multilevel Pain Assessment Using Facial Images: Systematic Review and Meta-Analysis

**DOI:** 10.2196/51250

**Published:** 2024-04-12

**Authors:** Jian Huo, Yan Yu, Wei Lin, Anmin Hu, Chaoran Wu

**Affiliations:** 1 Boston Intelligent Medical Research Center Shenzhen United Scheme Technology Company Limited Boston, MA United States; 2 Department of Anesthesia Shenzhen People's Hospital, The First Affiliated Hospital of Southern University of Science and Technology Shenzhen Key Medical Discipline Shenzhen China; 3 Shenzhen United Scheme Technology Company Limited Shenzhen China; 4 The Second Clinical Medical College Jinan University Shenzhen China

**Keywords:** computer vision, facial image, monitoring, multilevel pain assessment, pain, postoperative, status

## Abstract

**Background:**

The continuous monitoring and recording of patients’ pain status is a major problem in current research on postoperative pain management. In the large number of original or review articles focusing on different approaches for pain assessment, many researchers have investigated how computer vision (CV) can help by capturing facial expressions. However, there is a lack of proper comparison of results between studies to identify current research gaps.

**Objective:**

The purpose of this systematic review and meta-analysis was to investigate the diagnostic performance of artificial intelligence models for multilevel pain assessment from facial images.

**Methods:**

The PubMed, Embase, IEEE, Web of Science, and Cochrane Library databases were searched for related publications before September 30, 2023. Studies that used facial images alone to estimate multiple pain values were included in the systematic review. A study quality assessment was conducted using the Quality Assessment of Diagnostic Accuracy Studies, 2nd edition tool. The performance of these studies was assessed by metrics including sensitivity, specificity, log diagnostic odds ratio (LDOR), and area under the curve (AUC). The intermodal variability was assessed and presented by forest plots.

**Results:**

A total of 45 reports were included in the systematic review. The reported test accuracies ranged from 0.27-0.99, and the other metrics, including the mean standard error (MSE), mean absolute error (MAE), intraclass correlation coefficient (ICC), and Pearson correlation coefficient (PCC), ranged from 0.31-4.61, 0.24-2.8, 0.19-0.83, and 0.48-0.92, respectively. In total, 6 studies were included in the meta-analysis. Their combined sensitivity was 98% (95% CI 96%-99%), specificity was 98% (95% CI 97%-99%), LDOR was 7.99 (95% CI 6.73-9.31), and AUC was 0.99 (95% CI 0.99-1). The subgroup analysis showed that the diagnostic performance was acceptable, although imbalanced data were still emphasized as a major problem. All studies had at least one domain with a high risk of bias, and for 20% (9/45) of studies, there were no applicability concerns.

**Conclusions:**

This review summarizes recent evidence in automatic multilevel pain estimation from facial expressions and compared the test accuracy of results in a meta-analysis. Promising performance for pain estimation from facial images was established by current CV algorithms. Weaknesses in current studies were also identified, suggesting that larger databases and metrics evaluating multiclass classification performance could improve future studies.

**Trial Registration:**

PROSPERO CRD42023418181; https://www.crd.york.ac.uk/prospero/display_record.php?RecordID=418181

## Introduction

The definition of pain was revised to “an unpleasant sensory and emotional experience associated with, or resembling that associated with, actual or potential tissue damage” in 2020 [1]. Acute postoperative pain management is important, as pain intensity and duration are critical influencing factors for the transition of acute pain to chronic postsurgical pain [2]. To avoid the development of chronic pain, guidelines were promoted and discussed to ensure safe and adequate pain relief for patients, and clinicians were recommended to use a validated pain assessment tool to track patients’ responses [3]. However, these tools, to some extent, depend on communication between physicians and patients, and continuous data cannot be provided [4]. The continuous assessment and recording of patient pain intensity will not only reduce caregiver burden but also provide data for chronic pain research. Therefore, automatic and accurate pain measurements are necessary.

Researchers have proposed different approaches to measuring pain intensity. Physiological signals, for example, electroencephalography and electromyography, have been used to estimate pain [5-7]. However, it was reported that current pain assessment from physiological signals has difficulties isolating stress and pain with machine learning techniques, as they share conceptual and physiological similarities [8]. Recent studies have also investigated pain assessment tools for certain patient subgroups. For example, people with deafness or an intellectual disability may not be able to communicate well with nurses, and an objective pain evaluation would be a better option [9,10]. Measuring pain intensity from patient behaviors, such as facial expressions, is also promising for most patients [4]. As the most comfortable and convenient method, computer vision techniques require no attachments to patients and can monitor multiple participants using 1 device [4]. However, pain intensity, which is important for pain research, is often not reported.

With the growing trend of assessing pain intensity using artificial intelligence (AI), it is necessary to summarize current publications to determine the strengths and gaps of current studies. Existing research has reviewed machine learning applications for acute postoperative pain prediction, continuous pain detection, and pain intensity estimation [10-14]. Input modalities, including facial recordings and physiological signals such as electroencephalography and electromyography, were also reviewed [5,8]. There have also been studies focusing on deep learning approaches [11]. AI was applied in children and infant pain evaluation as well [15,16]. However, no study has focused on pain intensity measurement, and no comparison of test accuracy results has been made.

Current AI applications in pain research can be categorized into 3 types: pain assessment, pain prediction and decision support, and pain self-management [14]. We consider accurate and automatic pain assessment to be the most important area and the foundation of future pain research. In this study, we performed a systematic review and meta-analysis to assess the diagnostic performance of current publications for multilevel pain evaluation.

## Methods

This study was registered with PROSPERO (International Prospective Register of Systematic Reviews; CRD42023418181) and carried out strictly following the PRISMA (Preferred Reporting Items for Systematic Reviews and Meta-Analyses) guidelines [17]**.**

### Study Eligibility

Studies that reported AI techniques for multiclass pain intensity classification were eligible. Records including nonhuman or infant participants or 2-class pain detection were excluded. Only studies using facial images of the test participants were accepted. Clinically used pain assessment tools, such as the visual analog scale (VAS) and numerical rating scale (NRS), and other pain intensity indicators, were rejected in the meta-analysis. Textbox 1 presents the eligibility criteria.

**Study eligibility criteria.**

**Study characteristics and inclusion criteria**

- Participants: children and adults aged 12 months or older
- Setting: no restrictions
- Index test: artificial intelligence models that measure pain intensity from facial images
- Reference standard: no restrictions for systematic review; Prkachin and Solomon pain intensity score for meta-analysis
- Study design: no need to specify

**Study characteristics and exclusion criteria**

- Participants: infants aged 12 months or younger and animal subjects
- Setting: no need to specify
- Index test: studies that use other information such as physiological signals
- Reference standard: other pain evaluation tools, e.g., NRS, VAS, were excluded from meta-analysis
- Study design: reviews

**Report characteristics and inclusion criteria**

- Year: published between January 1, 2012, and September 30, 2023
- Language: English only
- Publication status: published
- Test accuracy metrics: no restrictions for systematic reviews; studies that reported contingency tables were included for meta-analysis

**Report characteristics and exclusion criteria**

- Year: no need to specify
- Language: no need to specify
- Publication status: preprints not accepted
- Test accuracy metrics: studies that reported insufficient metrics were excluded from meta-analysis

### Search Strategy

In this systematic review, databases including PubMed, Embase, IEEE, Web of Science, and the Cochrane Library were searched until December 2022, and no restrictions were applied. Keywords were “artificial intelligence” AND “pain recognition.” Multimedia Appendix 1 shows the detailed search strategy.

### Data Extraction

A total of 2 viewers screened titles and abstracts and selected eligible records independently to assess eligibility, and disagreements were solved by discussion with a third collaborator. A consentient data extraction sheet was prespecified and used to summarize study characteristics independently. Table S5 in Multimedia Appendix 1 shows the detailed items and explanations for data extraction. Diagnostic accuracy data were extracted into contingency tables, including true positives, false positives, false negatives, and true negatives. The data were used to calculate the pooled diagnostic performance of the different models. Some studies included multiple models, and these models were considered independent of each other.

### Study Quality Assessment

All included studies were independently assessed by 2 viewers using the Quality Assessment of Diagnostic Accuracy Studies 2 (QUADAS-2) tool [18]. QUADAS-2 assesses bias risk across 4 domains, which are patient selection, index test, reference standard, and flow and timing. The first 3 domains are also assessed for applicability concerns. In the systematic review, a specific extension of QUADAS-2, namely, QUADAS-AI, was used to specify the signaling questions [19].

### Meta-Analysis

Meta-analyses were conducted between different AI models. Models with different algorithms or training data were considered different. To evaluate the performance differences between models, the contingency tables during model validation were extracted. Studies that did not report enough diagnostic accuracy data were excluded from meta-analysis.

Hierarchical summary receiver operating characteristic (SROC) curves were fitted to evaluate the diagnostic performance of AI models. These curves were plotted with 95% CIs and prediction regions around averaged sensitivity, specificity, and area under the curve estimates. Heterogeneity was assessed visually by forest plots. A funnel plot was constructed to evaluate the risk of bias.

Subgroup meta-analyses were conducted to evaluate the performance differences at both the model level and task level, and subgroups were created based on different tasks and the proportion of positive and negative samples.

All statistical analyses and plots were produced using RStudio (version 4.2.2; R Core Team) and the R package *meta4diag* (version 2.1.1; Guo J and Riebler A) [20].

## Results

### Study Selection and Included Study Characteristics

**Figure 1:**
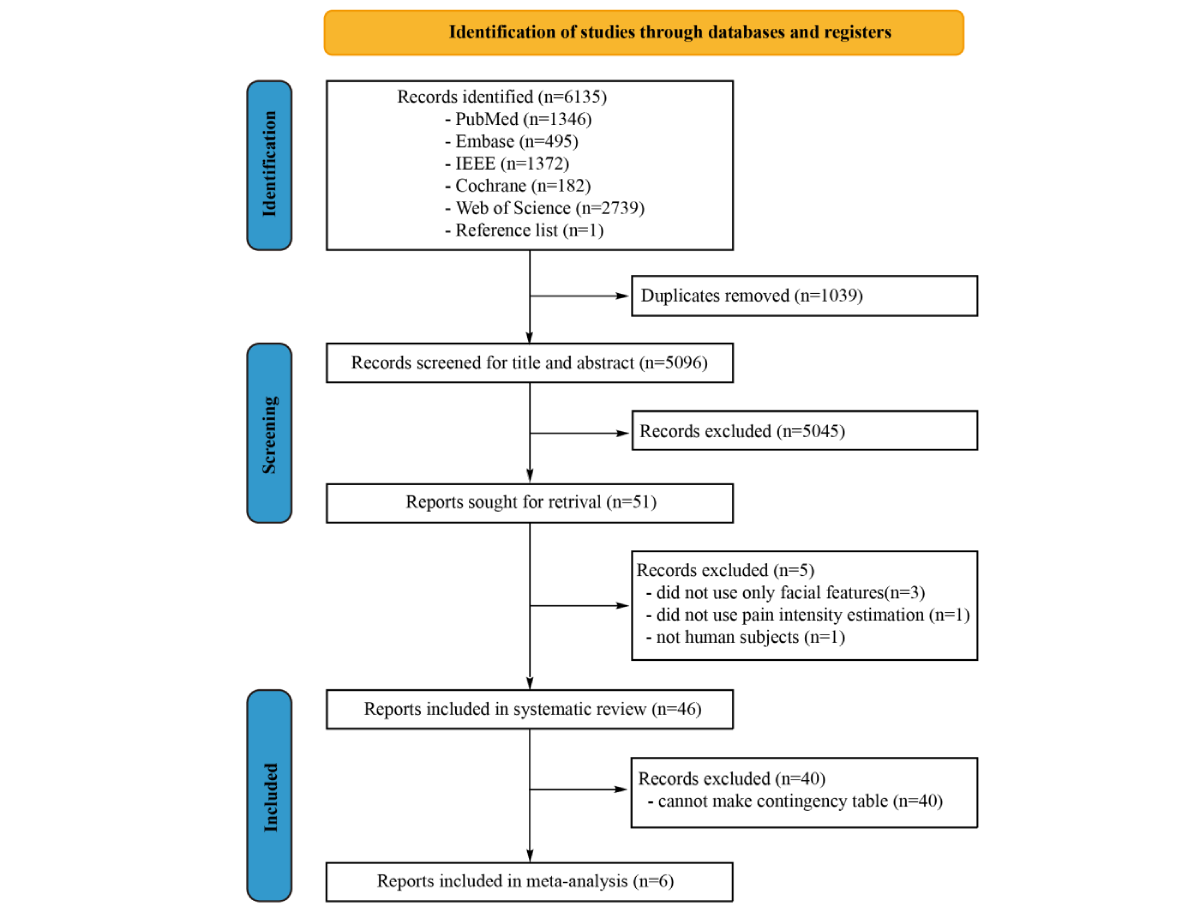
Preferred Reporting Items for Systematic Review and Meta-Analysis (PRISMA) flowchart of study selection.

A flow diagram representing the study selection process is shown in (Figure 1). After removing 1039 duplicates, the titles and abstracts of a total of 5653 papers were screened, and the percentage agreement of title or abstract screening was 97%. After screening, 51 full-text reports were assessed for eligibility, among which 45 reports were included in the systematic review [21-65]. The percentage agreement of the full-text review was 87%. In 40 of the included studies, contingency tables could not be made. Meta-analyses were conducted based on 8 AI models extracted from 6 studies. Individual study characteristics included in the systematic review are provided in Tables 1 and 2. The facial feature extraction method can be categorized into 2 classes: geometrical features (GFs) and deep features (DFs). One typical method of extracting GFs is to calculate the distance between facial landmarks. DFs are usually extracted by convolution operations. A total of 20 studies included temporal information, but most of them (18) extracted temporal information through the 3D convolution of video sequences. Feature transformation was also commonly applied to reduce the time for training or fuse features extracted by different methods before inputting them into the classifier. For classifiers, support vector machines (SVMs) and convolutional neural networks (CNNs) were mostly used. Table 1 presents the model designs of the included studies.

**Table 1:** Model designs of studies included in the systematic review.

Author and year	Facial feature descriptor	Temporal features^a^	Feature transformation	Classification method
Hammal and Cohn (2012) [21]	CAPP	–	Log-normal filters	SVM^b^
Adibuzzaman et al (2015) [22]	PCA	–	None	Euclidean distance; angular distance; SVM
Majumder et al (2015) [23]	GF^c^; DDF	+	None	GMM^d^; SVM
Rathee and Ganotra (2015) [24]	TPS^e^	+	DML^f^	SVM
Sikka et al (2015) [25]	CERT	++	None	Linear regression
Rathee and Ganotra (2016) [26]	Gabor; LBP; HOG	–	MDML^g^	SVM
Zhou et al (2016) [27]	AAM^h^	++	Flattening	RCNN
Egede et al (2017) [28]	GF; HOG; CNN;	++	RVR^i^	RVR
Martinez et al (2017) [29]	PSPI^j^; I-FES^k^;	++	None	LSTM^l^-RNN; RNN-HCRF^m^
Bourou et al (2018) [30]	GF; Color	–	Statistical metrics	GLMM^n^
Haque et al (2018) [31]	CNN-RGB	++	Fine-tuned VGGFace	CNN
Semwal et al (2018) [32]	2D-Conv	–	Maxpooling	CNN
Tavakolian and Hadid (2018) [33]	Pretrained CASIA	++	VLAD^o^	BE
Tavakolian and Hadid (2018) [34]	3D-convolution	++	Average pooling	CNN
Wang and Sun (2018) [35]	3D-convolution; HOG; DFGS	++	SVR^p^	SVR
Bargshady et al (2019) [36]	Fine-tuned VGGFace	–	None	RNN
Casti et al (2019) [37]	LBP	–	MDS^q^	CNN
Lee and Wang (2019) [38]	CNN-RGB	–	None	ELM^r^
Saha et al (2019) [39]	PCA	–	None	NR
Tavakolian and Hadid (2019) [40]	3D-convolution	++	None	CNN
Bargshady et al (2020) [41]	VGGFace	–	PCA;DNN	EDLM^s^
Bargshady et al (2020) [42]	VGGFace	–	PCA	EDLM^s^
Dragomir et al (2020) [43]	ResNet	–	None	ResNet
Huang et al (2020) [44]	AAM	++	RNN-GRU	SVM
Mallol-Ragolta et al (2020) [45]	GF; HOG; HOG; OpenFace; VGGFace; ResNet-50	–	None	LSTM-RNN
Peng et al (2020) [46]	DCNN^t^	–	Probabilistic combination	Multiscale deep fusion network
Tavakolian et al (2020) [47]	GSM^u^	++	Aggregation	SNN
Xu and de Sa (2020) [48]	Handcrafted	++	None	NN
Pikulkaew et al (2021) [49]	None	–	None	DCNN
Rezaei et al (2021) [50]	CNN	++	Flattening	NN
Semwal and Londhe (2021) [51]	CNN	–	None	CNN
Semwal and Londhe (2021) [52]	VGGNet; MobileNet; GoogLeNet	–	None	CNN
Szczapa et al (2021) [53]	Landmark trajectory	++	None	SVR
Ting et al (2021) [54]	None	++	DOML^v^	NN
Xin et al (2021) [55]	CNN	++	None	LIAN^w^
Alghamdi and Alaghband (2022) [56]	OpenCV	–	Flattening	Shallow CNN
Barua et al (2022) [57]	P-DarkNet19	–	INCA	k-NN
Fontaine et al (2022) [58]	OpenCV	–	None	CNN; SVM
Hosseini et al 2022) [59]	Convolution	–	None	DCNN
Huang et al (2022) [60]	3D-CNN (S3D-G)	++	None	CNN
Islamadina et al (2022) [61]	CNN	–	None	CNN
Swetha et al (2022) [62]	None	–	None	CNN
Wu et al (2022) [63]	CNN	+	Siamese network; BiLSTM^x^	NN
Ismail and Waseem (2023) [64]	CNN	–	None	CNN
Vu and Beurton-Aimar (2023) [65]	CNN	–	Average pooling	LSTM network

^a^No temporal features are shown by – symbol, time information extracted from 2 images at different time by +, and deep temporal features extracted through the convolution of video sequences by ++.

^b^SVM: support vector machine.

^c^GF: geometric feature.

^d^GMM: gaussian mixture model.

^e^TPS: thin plate spline.

^f^DML: distance metric learning.

^g^MDML: multiview distance metric learning.

^h^AAM: active appearance model.

^i^RVR: relevance vector regressor.

^j^PSPI: Prkachin and Solomon pain intensity.

^k^I-FES: individual facial expressiveness score.

^l^LSTM: long short-term memory.

^m^HCRF: hidden conditional random field.

^n^GLMM: generalized linear mixed model.

^o^VLAD: vector of locally aggregated descriptor.

^p^SVR: support vector regression.

^q^MDS: multidimensional scaling.

^r^ELM: extreme learning machine.

^s^Labeled to distinguish different architectures of ensembled deep learning models.

^t^DCNN: deep convolutional neural network.

^u^GSM: gaussian scale mixture.

^v^DOML: distance ordering metric learning.

^w^LIAN: locality and identity aware network.

^x^BiLSTM: bidirectional long short-term memory.

**Table 2:** Characteristics of model training and validation.

Author and year	Database	Objects	Output levels	Validation method	External validation	Evaluation metrics
Hammal and Cohn (2012) [21]	UNBC^a^	Frame	4	5-fold; LOSO^b^	No	ICC^c^ 0.85, 0.55; *F*_1_-score 0.96, 0.67
Adibuzzaman et al (2015) [22]	Self-prepared	Image	3	10-fold	Yes	Sensitivity 0.53; Specificity 0.7
Majumder et al (2015) [23]	UNBC	Frame	16	5-fold	No	Accuracy 87.43
Rathee and Ganotra (2015) [24]	UNBC	Frame	16	LOO; 10-fold	No	Accuracy 0.96; CT^d^
Sikka et al (2015) [25]	Self-prepared	Sequence	11	LOSO	No	AUC^e^ 0.94; Cohen κ0.61
Rathee and Ganotra (2016) [26]	UNBC	Frame	4	5-fold	No	Accuracy 0.75
Zhou et al (2016) [27]	UNBC	Frame	16	LOSO	No	MSE^f^ 1.54; PCC^g^ 0.65
Egede et al (2017) [28]	UNBC	Frame	16	LOSO	No	RMSE^h^<1; PCC 0.67
Martinez et al (2017) [29]	UNBC	Sequence	11	Split	No	MAE^i^ 2.8; ICC^j^ 0.19
Bourou et al (2018) [30]	BioVid	Frame	5	10-fold	No	Accuracy 0.27; RCI 0.03
Haque et al (2018) [31]	MIntPain	Frame	5	5-fold	No	CT
Semwal and Londhe (2018) [32]	UNBC	Frame	3	Split	No	CT; Accuracy 0.93
Tavakolian and Hadid (2018) [33]	UNBC	Frame	16	LOSO	No	MSE 0.69; PCC 0.81
Tavakolian and Hadid (2018) [34]	UNBC	Frame	16	LOSO	No	MSE 0.53; ICC 0.75; PCC 0.84
Wang and Sun (2018) [35]	UNBC	Frame	16	LOSO	No	RMSE 0.94; PCC 0.68
Bargshady et al (2019) [36]	UNBC	Frame	4	LOSO	No	Accuracy 0.75; AUC 0.83; MSE 0.95
Casti et al (2019) [37]	UNBC	Frame	11	Split	No	Recall 0.92; Precision 0.82
Lee and Wang (2019) [38]	UNBC	Frame	16	5-fold	No	MSE 1.22; PCC 0.5
Saha et al (2019) [39]	Self-prepared	Image	3	10-fold	No	Accuracy 0.71; CT
Tavakolian and Hadid (2019) [40]	UNBC	Frame	5; 16	LOSO	No	MSE 0.32; PCC 0.92; AUC 0.86
Bargshady et al (2020) [41]	MIntPain; UNBC	Frame	5	10-fold	No	Accuracy 0.89; AUC 0.93
Bargshady et al (2020) [42]	UNBC	Frame	4	10-fold	No	Accuracy 0.91; AUC 0.98
Dragomir et al (2020) [43]	BioVid	Frame	5	CV	No	Accuracy 36.6
Huang et al (2020) [44]	UNBC	Frame	6	Split	No	PCC 0.89; ICC 0.72; MSE 0.21; MAE 0.24
Mallol-Ragolta et al (2020) [45]	EmoPain	Frame	11	Split	No	CCC^k^ 0.174
Peng et al (2020) [46]	UNBC	Frame	5	NR	No	Accuracy 0.80; PCC 0.6; MAE 0.57; MSE 0.82
Tavakolian et al (2020) [47]	BioVid; UNBC	Frame	5; 16	LOSO	Yes	MSE 1.03, 0.92; AUC 0.69, 0.71
Xu and de Sa (2020) [48]	UNBC	Sequence	6; 11; 16; 16	5-fold	No	MSE 4.61; MAE 1.73; ICC 0.61; PCC 0.67
Pikulkaew et al (2021) [49]	UNBC	Frame	3	NR	No	Accuracy 0.93
Rezaei et al (2021) [50]	UofR; UNBC	Frame	16	5-fold	Yes	PCC 0.48-0.7; ICC 0.31-0.59^l^
Semwal and Londhe (2021) [51]	Self-prepared	Frame	4	5-fold	No	CT; Accuracy 0.97
Semwal and Londhe (2021) [52]	UNBC	Frame	5	10-fold	No	CT; *F*_1_-score 0.91
Szczapa et al (2021) [53]	UNBC	Sequence	11	5-fold; LOO; LOSO	No	MAE 2.44; RMSE 3.15
Ting et al (2021) [54]	UNBC	Sequence	11	5-fold; LOSO	No	MAE 1.62; MSE 4.39; ICC 0.66
Xin et al (2021) [55]	UNBC	Frame	4	LOSO	No	Accuracy 0.89; ICC 0.61; PCC 0.81; MAE 0.45; MSE 0.66
Alghamdi and Alaghband (2022) [56]	UNBC	Frame	4	Split	No	Accuracy 0.99
Barua et al (2022) [57]	DISFA; UNBC	Frame	4	10-fold	No	CT; Accuracy 0.95
Fontaine et al (2022) [58]	Self-prepared	Frame	4	Split	No	Sensitivity 0.90
Hosseini et al (2022) [59]	UNBC	Frame	7	NR	No	Accuracy 0.85; AUC 0.88; PCC 0.83
Huang et al (2022) [60]	UNBC	Frame	16	LOSO	No	MAE 0.4; MSE 0.76; PCC 0.82
Islamadina et al (2022) [61]	MIntPian	Frame	5	CV	No	CT; Accuracy 1.0
Swetha et al (2022) [62]	Self-prepared	Frame	4	NR	No	Accuracy 0.75
Wu et al (2022) [63]	Self-prepared	Frame; sequence	3	Split	No	Accuracy 0.81
Ismail and Waseem 2023 [64]	UNBC	Frame	16	5-fold	No	MAE 0.36; MSE 1.73; Accuracy 0.82
Vu and Beurton-Aimar 2023 [65]	DISFA; UNBC	Frame	16	LOSO	No	MSE 0.57; MAE 0.35; ICC 0.83; PCC 0.81

^a^UNBC: University of Northern British Columbia-McMaster shoulder pain expression archive database.

^b^LOSO: leave one subject out cross-validation.

^c^ICC: intraclass correlation coefficient.

^d^CT: contingency table.

^e^AUC: area under the curve.

^f^MSE: mean standard error.

^g^PCC: Pearson correlation coefficient.

^h^RMSE: root mean standard error.

^i^MAE: mean absolute error.

^j^ICC: intraclass coefficient.

^k^CCC: concordance correlation coefficient.

^l^Reported both external and internal validation results and summarized as intervals.

Table 2 summarizes the characteristics of model training and validation. Most studies used publicly available databases, for example, the University of Northern British Columbia-McMaster shoulder pain expression archive database [57]. Table S4 in Multimedia Appendix 1 summarizes the public databases. A total of 7 studies used self-prepared databases. Frames from video sequences were the most used test objects, as 37 studies output frame-level pain intensity, while few measure pain intensity from video sequences or photos. It was common that a study redefined pain levels to have fewer classes than ground-truth labels. For model validation, cross-validation and leave-one-subject-out validation were commonly used. Only 3 studies performed external validation. For reporting test accuracies, different evaluation metrics were used, including sensitivity, specificity, mean absolute error (MAE), mean standard error (MSE), Pearson correlation coefficient (PCC), and intraclass coefficient (ICC).

### Methodological Quality of Included Studies

**Figure 2:**
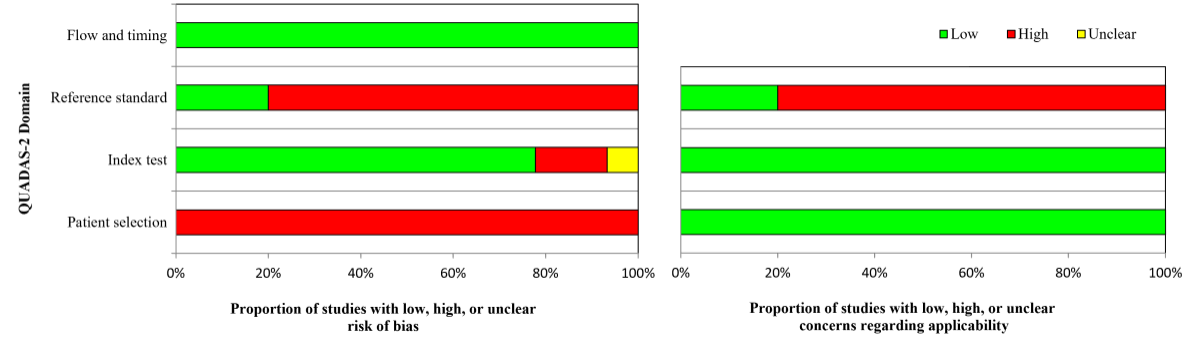
Risk of bias and applicability proportions. QUADAS-2: Quality Assessment of Diagnostic Accuracy Studies 2.

Table S2 in Multimedia Appendix 1 presents the study quality summary, as assessed by QUADAS-2. There was a risk of bias in all studies, specifically in terms of patient selection, caused by 2 issues. First, the training data are highly imbalanced, and any method to adjust the data distribution may introduce bias. Next, the QUADAS-AI correspondence letter [19] specifies that preprocessing of images that changes the image size or resolution may introduce bias. However, the applicability concern is low, as the images properly represent the feeling of pain. Studies that used cross-fold validation or leave-one-out cross-validation were considered to have a low risk of bias. Although the Prkachin and Solomon pain intensity (PSPI) score was used by most of the studies, its ability to represent individual pain levels was not clinically validated; as such, the risk of bias and applicability concerns were considered high when the PSPI score was used as the index test. As an advantage of computer vision techniques, the time interval between the index tests was short and was assessed as having a low risk of bias. Risk proportions are shown in Figure 2. For all 315 entries, 39% (124) were assessed as high-risk. In total, 5 studies had the lowest risk of bias, with 6 domains assessed as low risk [26,27,31,32,59].

### Pooled Performance of Included Models

**Figure 3:**
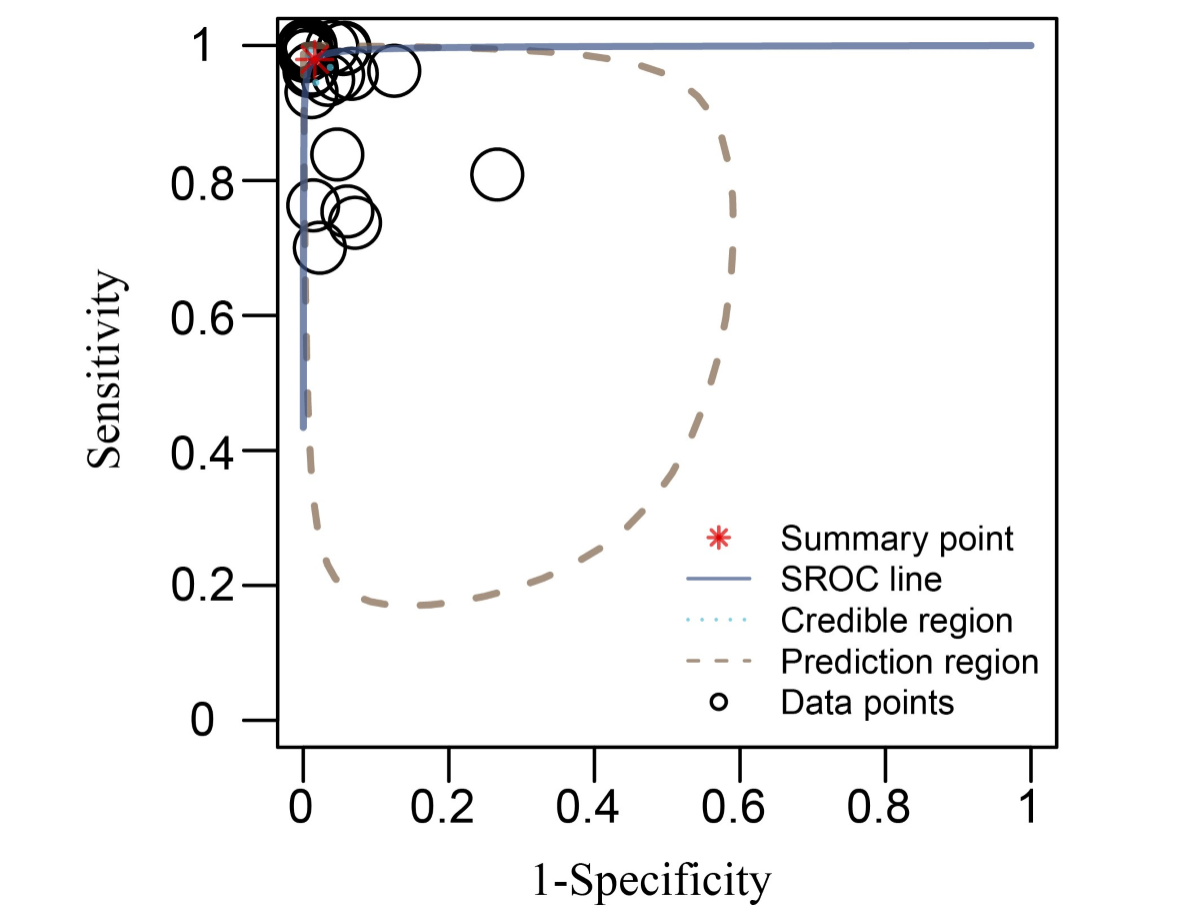
Summary receiver operating characteristic (SROC) curve plots of the summarized results.

In 6 studies included in the meta-analysis, there were 8 different models. The characteristics of these models are summarized in Table S1 in Multimedia Appendix 2 [23,24,26,32,41,57]. Classification of PSPI scores greater than 0, 2, 3, 6, and 9 was selected and considered as different tasks to create contingency tables. The test performance is shown in Figure 3 as hierarchical SROC curves; 27 contingency tables were extracted from 8 models. The sensitivity, specificity, and LDOR were calculated, and the combined sensitivity was 98% (95% CI 96%-99%), the specificity was 98% (95% CI 97%-99%), the LDOR was 7.99 (95% CI 6.73-9.31) and the AUC was 0.99 (95% CI 0.99-1).

### Subgroup Analysis

In this study, subgroup analysis was conducted to investigate the performance differences within models. A total of 8 models were separated and summarized as a forest plot in Multimedia Appendix 3 [23,24,26,32,41,57]. For model 1, the pooled sensitivity, specificity, and LDOR were 95% (95% CI 86%-99%), 99% (95% CI 98%-100%), and 8.38 (95% CI 6.09-11.19), respectively. For model 2, the pooled sensitivity, specificity, and LDOR were 94% (95% CI 84%-99%), 95% (95% CI 88%-99%), and 6.23 (95% CI 3.52-9.04), respectively. For model 3, the pooled sensitivity, specificity, and LDOR were 100% (95% CI 99%-100%), 100% (95% CI 99%-100%), and 11.55% (95% CI 8.82-14.43), respectively. For model 4, the pooled sensitivity, specificity, and LDOR were 83% (95% CI 43%-99%), 94% (95% CI 79%-99%), and 5.14 (95% CI 0.93-9.31), respectively. For model 5, the pooled sensitivity, specificity, and LDOR were 92% (95% CI 68%-99%), 94% (95% CI 78%-99%), and 6.12 (95% CI 1.82-10.16), respectively. For model 6, the pooled sensitivity, specificity, and LDOR were 94% (95% CI 74%-100%), 94% (95% CI 78%-99%), and 6.59 (95% CI 2.21-11.13), respectively. For model 7, the pooled sensitivity, specificity, and LDOR were 98% (95% CI 90%-100%), 97% (95% CI 87%-100%), and 8.31 (95% CI 4.3-12.29), respectively. For model 8, the pooled sensitivity, specificity, and LDOR were 98% (95% CI 93%-100%), 97% (95% CI 88%-100%), and 8.65 (95% CI 4.84-12.67), respectively.

### Heterogeneity Analysis

**Figure 4:**
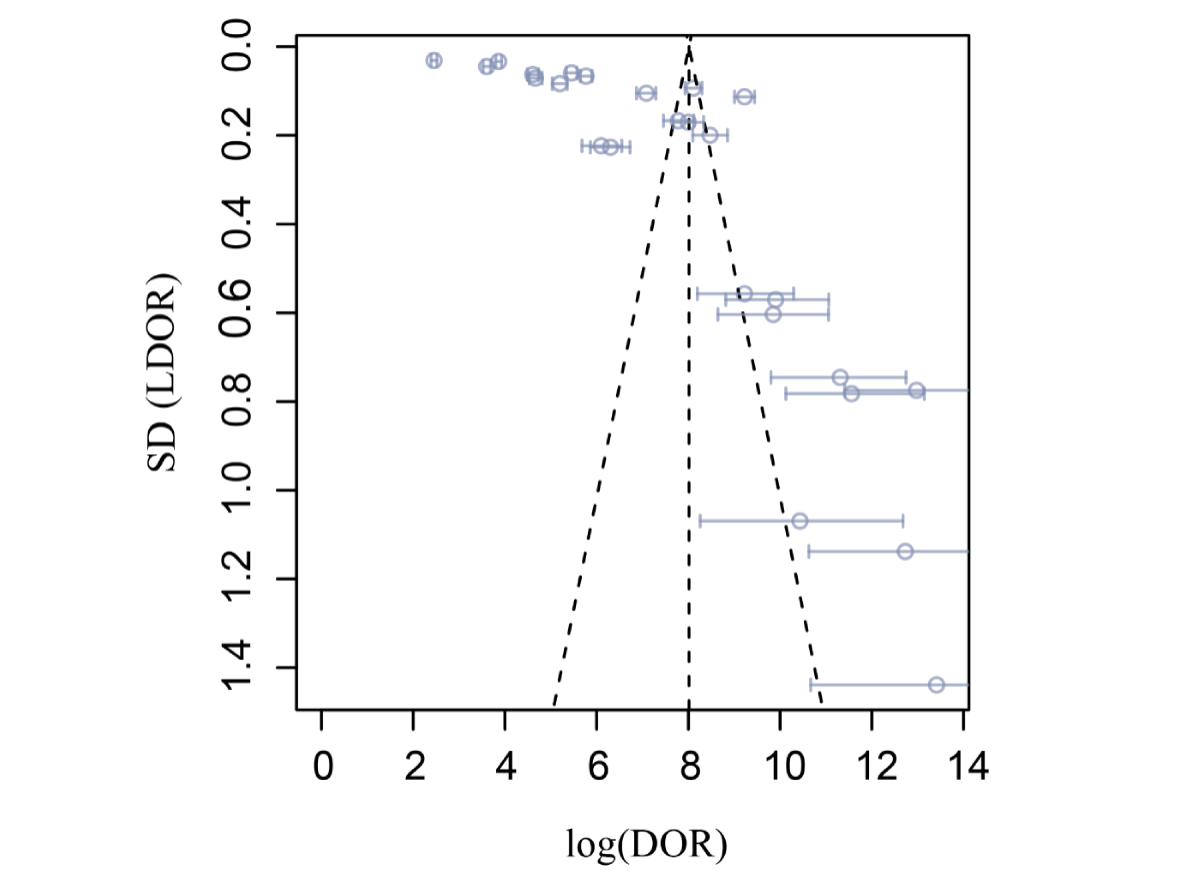
In the funnel plot of the test results, significant heterogeneity was observed. DOR: diagnostic odds ratio; LDOR: log diagnostic odds ratio.

The meta-analysis results indicated that AI models are applicable for estimating pain intensity from facial images. However, extreme heterogeneity existed within the models except for models 3 and 5, which were proposed by Rathee and Ganotra [24] and Semwal and Londhe [32]. A funnel plot is presented in Figure 4. A high risk of bias was observed.

## Discussion

Pain management has long been a critical problem in clinical practice, and the use of AI may be a solution. For acute pain management, automatic measurement of pain can reduce the burden on caregivers and provide timely warnings. For chronic pain management, as specified by Glare et al [2], further research is needed, and measurements of pain presence, intensity, and quality are one of the issues to be solved for chronic pain studies. Computer vision could improve pain monitoring through real-time detection for clinical use and data recording for prospective pain studies. To our knowledge, this is the first meta-analysis dedicated to AI performance in multilevel pain level classification.

In this study, one model’s performance at specific pain levels was described by stacking multiple classes into one to make each task a binary classification problem. After careful selection in both the medical and engineering databases, we observed promising results of AI in evaluating multilevel pain intensity through facial images, with high sensitivity (98%), specificity (98%), LDOR (7.99), and AUC (0.99). It is reasonable to believe that AI can accurately evaluate pain intensity from facial images. Moreover, the study quality and risk of bias were evaluated using an adapted QUADAS-2 assessment tool, which is a strength of this study.

To investigate the source of heterogeneity, it was assumed that a well-designed model should have familiar size effects regarding different levels, and a subgroup meta-analysis was conducted. The funnel and forest plots exhibited extreme heterogeneity. The model’s performance at specific pain levels was described and summarized by a forest plot. Within-model heterogeneity was observed in Multimedia Appendix 3 [23,24,26,32,41,57] except for 2 models. Models 3 and 5 were different in many aspects, including their algorithms and validation methods, but were both trained with a relatively small data set, and the proportion of positive and negative classes was relatively close to 1. Because training with imbalanced data is a critical problem in computer vision studies [66], for example, in the University of Northern British Columbia-McMaster pain data set, fewer than 10 frames out of 48,398 had a PSPI score greater than 13. Here, we emphasized that imbalanced data sets are one major cause of heterogeneity, resulting in the poorer performance of AI algorithms.

We tentatively propose a method to minimize the effect of training with imbalanced data by stacking multiple classes into one class, which is already presented in studies included in the systematic review [26,32,42,57]. Common methods to minimize bias include resampling and data augmentation [66]. This proposed method is used in the meta-analysis to compare the test results of different studies as well. The stacking method is available when classes are only different in intensity. A disadvantage of combined classes is that the model would be insufficient in clinical practice when the number of classes is low. Commonly used pain evaluation tools, such as VAS, have 10 discrete levels. It is recommended that future studies set the number of pain levels to be at least 10 for model training.

This study is limited for several reasons. First, insufficient data were included because different performance metrics (mean standard error and mean average error) were used in most studies, which could not be summarized into a contingency table. To create a contingency table that can be included in a meta-analysis, the study should report the following: the number of objects used in each pain class for model validation, and the accuracy, sensitivity, specificity, and *F*_1_-score for each pain class. This table cannot be created if a study reports the MAE, PCC, and other commonly used metrics in AI development. Second, a small study effect was observed in the funnel plot, and the heterogeneity could not be minimized. Another limitation is that the PSPI score is not clinically validated and is not the only tool that assesses pain from facial expressions. There are other clinically validated pain intensity assessment methods, such as the Faces Pain Scale-revised, Wong-Baker Faces Pain Rating Scale, and Oucher Scale [3]. More databases could be created based on the above-mentioned tools. Finally, AI-assisted pain assessments were supposed to cover larger populations, including incommunicable patients, for example, patients with dementia or patients with masked faces. However, only 1 study considered patients with dementia, which was also caused by limited databases [50].

AI is a promising tool that can help in pain research in the future. In this systematic review and meta-analysis, one approach using computer vision was investigated to measure pain intensity from facial images. Despite some risk of bias and applicability concerns, CV models can achieve excellent test accuracy. Finally, more CV studies in pain estimation, reporting accuracy in contingency tables, and more pain databases are encouraged for future studies. Specifically, the creation of a balanced public database that contains not only healthy but also nonhealthy participants should be prioritized. The recording process would be better in a clinical environment. Then, it is recommended that researchers report the validation results in terms of accuracy, sensitivity, specificity, or contingency tables, as well as the number of objects for each pain class, for the inclusion of a meta-analysis.

## Appendix

Multimedia Appendix 1 PRISMA checklist, risk of bias summary, search strategy, database summary and reported items and explanations.

Multimedia Appendix 2 Study performance summary.

Multimedia Appendix 3 Forest plot presenting pooled performance of subgroups in meta-analysis.

## Abbreviations

AI: artificial intelligence
AUC: area under the curve
CNN: convolutional neural network
CV: computer vision
DF: deep feature
GF: geometrical feature
ICC: intraclass correlation coefficient
LDOR: log diagnostic odds ratio
MAE: mean absolute error
NRS: numerical rating scale
PCC: Pearson correlation coefficient
PRISMA: Preferred Reporting Items for Systematic Review and Meta-Analysis
PROSPERO: International Prospective Register of Systematic Reviews
PSPI: Prkachin and Solomon pain intensity
QUADAS-2: Quality Assessment of Diagnostic Accuracy Studies 2
SROC: summary receiver operating characteristic
SVM: support vector machine
VAS: visual analog scale

## Data Availability

The data sets generated during and analyzed during this study are available in the Figshare repository [67].
