# Correction: Application of AI in Multilevel Pain Assessment Using Facial Images: Systematic Review and Meta-Analysis

**DOI:** 10.2196/59628

**Published:** 2024-04-17

**Authors:** Jian Huo, Yan Yu, Wei Lin, Anmin Hu, Chaoran Wu

**Affiliations:** 1 Boston Intelligent Medical Research Center Shenzhen United Scheme Technology Company Limited Boston, MA United States; 2 Department of Anesthesia Shenzhen People's Hospital, The First Affiliated Hospital of Southern University of Science and Technology Shenzhen Key Medical Discipline Shenzhen China; 3 Shenzhen United Scheme Technology Company Limited Shenzhen China; 4 The Second Clinical Medical College Jinan University Shenzhen China

In “Application of AI in Multilevel Pain Assessment Using Facial Images: Systematic Review and Meta-Analysis” (J Med Internet Res 2024;26:e51250) a redundant repetition was found.

The title was originally published as:

Application of AI in in Multilevel Pain Assessment Using Facial Images: Systematic Review and Meta-Analysis

It will now be changed to:

Application of AI in Multilevel Pain Assessment Using Facial Images: Systematic Review and Meta-Analysis

The correction will appear in the online version of the paper on the JMIR Publications website on April 17, 2024, together with the publication of this correction notice. Because this correction was made after submission to PubMed, PubMed Central, and other full-text repositories, the corrected article has also been resubmitted to those repositories.

