# Supplementary material for: Application of AI in Multilevel Pain Assessment Using Facial Images: Systematic Review and Meta-Analysis
Source: J Med Internet Res. 2024 Apr 12;26:e51250. doi: 10.2196/51250 (PMC11053395; doi:10.2196/51250)
Supplement: Multimedia Appendix 1 [file jmir_v26i1e51250_app1.doc]

Artificial intelligence application in multilevel pain assessment from facial images: A systematic review and meta-analysis.

**Table S1**. Preferred Reporting Items for Systematic Review and Meta-Analysis (PRISMA) checklist.

**Table S2**. Risk of bias and applicability concerns summary.

**Table S3**. Search strategy.

**Table S4**. Summary of public pain databases.

**Table S5**. Reported items and explanations.

**Table S1**. Preferred Reporting Items for Systematic Review and Meta-Analysis (PRISMA-DTA) checklist.

| Section/topic | # | PRISMA-DTA Checklist Item | Reported on page # |
| --- | --- | --- | --- |
| TITLE / ABSTRACT | | |  |
| Title | 1 | Identify the report as a systematic review (+/- meta-analysis) of diagnostic test accuracy (DTA) studies. | 1 |
| Abstract | 2 | Abstract: See PRISMA-DTA for abstracts. | 1 |
| INTRODUCTION | | |  |
| Rationale | 3 | Describe the rationale for the review in the context of what is already known. | 3 |
| Clinical role of index test | D1 | State the scientific and clinical background, including the intended use and clinical role of the index test, and if applicable, the rationale for minimally acceptable test accuracy (or minimum difference in accuracy for comparative design). | 3 |
| Objectives | 4 | Provide an explicit statement of question(s) being addressed in terms of participants, index test(s), and target condition(s). | 3 |
| METHODS | | |  |
| Protocol and registration | 5 | Indicate if a review protocol exists, if and where it can be accessed (e.g., Web address), and, if available, provide registration information including registration number. | 3 |
| Eligibility criteria | 6 | Specify study characteristics (participants, setting, index test(s), reference standard(s), target condition(s), and study design) and report characteristics (e.g., years considered, language, publication status) used as criteria for eligibility, giving rationale. | 4 |
| Information sources | 7 | Describe all information sources (e.g., databases with dates of coverage, contact with study authors to identify additional studies) in the search and date last searched. | 4 |
| Search | 8 | Present full search strategies for all electronic databases and other sources searched, including any limits used, such that they could be repeated. | Supplementary Table 3 |
| Study selection | 9 | State the process for selecting studies (i.e., screening, eligibility, included in systematic review, and, if applicable, included in the meta-analysis). | 6 |
| Data collection process | 10 | Describe method of data extraction from reports (e.g., piloted forms, independently, in duplicate) and any processes for obtaining and confirming data from investigators. | 3 |
| Definitions for data extraction | 11 | Provide definitions used in data extraction and classifications of target condition(s), index test(s), reference standard(s) and other characteristics (e.g. study design, clinical setting). | Supplementary Table 5 |
| Risk of bias and applicability | 12 | Describe methods used for assessing risk of bias in individual studies and concerns regarding the applicability to the review question. | 4 |
| Diagnostic accuracy measures | 13 | State the principal diagnostic accuracy measure(s) reported (e.g. sensitivity, specificity) and state the unit of assessment (e.g. per-patient, per-lesion). | 4 |
| Synthesis of results | 14 | Describe methods of handling data, combining results of studies and describing variability between studies. This could include, but is not limited to: a) handling of multiple definitions of target condition. b) handling of multiple thresholds of test positivity, c) handling multiple index test readers, d) handling of indeterminate test results, e) grouping and comparing tests, f) handling of different reference standards | 4 |
| Meta-analysis | D2 | Report the statistical methods used for meta-analyses, if performed. | 5 |
| Additional analyses | 16 | Describe methods of additional analyses (e.g., sensitivity or subgroup analyses, meta-regression), if done, indicating which were pre-specified. | 5 |
| RESULTS | | |  |
| Study selection | 17 | Provide numbers of studies screened, assessed for eligibility, included in the review (and included in meta-analysis, if applicable) with reasons for exclusions at each stage, ideally with a flow diagram. | 6 |
| Study characteristics | 18 | For each included study provide citations and present key characteristics including: a) participant characteristics (presentation, prior testing), b) clinical setting, c) study design, d) target condition definition, e) index test, f) reference standard, g) sample size, h) funding sources | 7-14 |
| Risk of bias and applicability | 19 | Present evaluation of risk of bias and concerns regarding applicability for each study. | 15 |
| Results of individual studies | 20 | For each analysis in each study (e.g. unique combination of index test, reference standard, and positivity threshold) report 2x2 data (TP, FP, FN, TN) with estimates of diagnostic accuracy and confidence intervals, ideally with a forest or receiver operator characteristic (ROC) plot. | Multimedia Appendix 2 |
| Synthesis of results | 21 | Describe test accuracy, including variability; if meta-analysis was done, include results and confidence intervals. | 15-16 |
| Additional analysis | 23 | Give results of additional analyses, if done (e.g., sensitivity or subgroup analyses, meta-regression; analysis of index test: failure rates, proportion of inconclusive results, adverse events). | 16-17 |
| DISCUSSION | | |  |
| Summary of evidence | 24 | Summarize the main findings including the strength of evidence. | 17 |
| Limitations | 25 | Discuss limitations from included studies (e.g. risk of bias and concerns regarding applicability) and from the review process (e.g. incomplete retrieval of identified research). | 18 |
| Conclusions | 26 | Provide a general interpretation of the results in the context of other evidence. Discuss implications for future research and clinical practice (e.g. the intended use and clinical role of the index test). | 18 |
| FUNDING | | |  |
| Funding | 27 | For the systematic review, describe the sources of funding and other support and the role of the funders. | 18 |
| *Adapted From:*  McInnes MDF, Moher D, Thombs BD, McGrath TA, Bossuyt PM, The PRISMA-DTA Group (2018). Preferred Reporting Items for a Systematic Review and Meta-analysis of Diagnostic Test Accuracy Studies: The PRISMA-DTA Statement. JAMA. 2018 Jan 23;319(4):388-396. doi: 10.1001/jama.2017.19163.  For more information, visit: www.prisma-statement.org. | | | |

**Table S2**. Risk of bias and applicability concerns summary.

| Study | Risk of Bias | | | |  | Applicability Concerns | | |
| --- | --- | --- | --- | --- | --- | --- | --- | --- |
|  | PATIENT SELECTION | INDEX TEST | REFERENCE STANDARD | FLOW AND TIMING |  | PATIENT SELECTION | INDEX TEST | REFERENCE STANDARD |
| Hammal et. al. 2012 | High | Low | High | Low |  | Low | Low | High |
| Adibuzzaman et. al. 2015 | High | Low | High | Low |  | Low | Low | High |
| Majumder et. al. 2015 | High | Low | High | Low |  | Low | Low | High |
| Rathee et. al. 2015 | High | Low | High | Low |  | Low | Low | High |
| Sikka et. al. 2015 | High | Low | High | Low |  | Low | Low | High |
| Rathee et. al. 2016 | High | Low | Low | Low |  | Low | Low | Low |
| zhou et. al. 2016 | High | Low | Low | Low |  | Low | Low | Low |
| Egede et. al. 2017 | High | Low | High | Low |  | Low | Low | High |
| Martinez et. al. 2017 | High | Low | High | Low |  | Low | Low | High |
| Bourou et. al. 2018 | High | High | High | Low |  | Low | Low | High |
| Haque et. al. 2018 | High | Low | Low | Low |  | Low | Low | Low |
| Semwal et. al. 2018 | High | Low | Low | Low |  | Low | Low | Low |
| Tavakolian et. al. 2018 | High | High | Low | Low |  | Low | Low | Low |
| Tavakolian et. al. 2018 | High | Low | High | Low |  | Low | Low | High |
| Wang et. al. 2018 | High | Low | High | Low |  | Low | Low | High |
| Bargshady et. al. 2019 | High | Low | High | Low |  | Low | Low | High |
| Casti et. al. 2019 | High | Low | High | Low |  | Low | Low | High |
| Lee et. al. 2019 | High | High | High | Low |  | Low | Low | High |
| Saha et. al. 2019 | High | Low | High | Low |  | Low | Low | High |
| Tavakolian et. al. 2019 | High | Low | High | Low |  | Low | Low | High |
| Bargshady et. al. 2020 | High | Low | High | Low |  | Low | Low | High |
| Bargshady et. al. 2020 | High | Low | Low | Low |  | Low | Low | Low |
| Dragomir et. al. 2020 | High | Low | High | Low |  | Low | Low | High |
| Huang et. al. 2020 | High | High | Low | Low |  | Low | Low | Low |
| Mallol-Ragolta et. al. 2020 | High | High | High | Low |  | Low | Low | High |
| Peng et. al. 2020 | High | Low | High | Low |  | Low | Low | High |
| Tavakolian et. al. 2020 | High | Unclear | High | Low |  | Low | Low | High |
| Xu et. al. 2020 | High | Low | High | Low |  | Low | Low | High |
| Pikulkaew et. al. 2021 | High | Low | High | Low |  | Low | Low | High |
| Rezaei et. al. 2021 | High | Low | High | Low |  | Low | Low | High |
| Semwal et. al. 2021 | High | Low | High | Low |  | Low | Low | High |
| Semwal et. al. 2021 | High | Low | High | Low |  | Low | Low | High |
| Szczapa et. al. 2021 | High | Low | High | Low |  | Low | Low | High |
| Ting et. al. 2021 | High | Low | High | Low |  | Low | Low | High |
| Xin et. al. 2021 | High | Low | High | Low |  | Low | Low | High |
| Alghamdi et. al. 2022 | High | Low | High | Low |  | Low | Low | High |
| Barua et. al. 2022 | High | Low | High | Low |  | Low | Low | High |
| Fontaine et. al. 2022 | High | Low | High | Low |  | Low | Low | High |
| Hosseini et. al. 2022 | High | Low | Low | Low |  | Low | Low | Low |
| Huang et. al. 2022 | High | Unclear | High | Low |  | Low | Low | High |
| Islamadina et. al. 2022 | High | Low | High | Low |  | Low | Low | High |
| Swetha et. al. 2022 | High | Unclear | Low | Low |  | Low | Low | Low |
| Wu et. al. 2022 | High | High | High | Low |  | Low | Low | High |
| Vu et. al. 2023 | High | High | High | Low |  | Low | Low | High |
| Ismail et. al. 2023 | High | Low | High | Low |  | Low | Low | High |

**Table S3**. Search strategy.

| Database | Search terms |
| --- | --- |
| PubMed | ((artificial intelligence[Title/Abstract]) OR (machine learning[Title/Abstract]) OR (deep learning[Title/Abstract]) OR (computer vision[Title/Abstract])) AND ((pain[Title/Abstract]) OR (pain management[Title/Abstract]) OR (pain assessment[Title/Abstract]) OR (pain recognition[Title/Abstract])) |
| Embase | 'artificial intelligence'/exp AND ('pain':ab OR 'pain management':ab OR 'pain assessment':ab OR 'pain recognition':ab) AND [2012-2022]/py |
| IEEEXplore | (("All Metadata":"artificial intelligence" OR "All Metadata":"learning" OR "All Metadata":"Model") AND ("All Metadata":"pain" OR "All Metadata":"pain assessment" OR "All Metadata":"pain management" OR "All Metadata":"pain recognition)) |
| Web of Science | (ALL=(artificial intelligence) OR ALL=(machine learning) OR ALL=(deep learning)) AND (TS=(pain) OR TS=(pain management) OR TS=(pain assessment) OR TS=(pain recognition)) |
| Cochrane | #1 ("artificial intelligence"):ti,ab,kw OR ("machine learning"):ti,ab,kw OR ("deep learning"):ti,ab,kw  #2 ("pain"):ti,ab,kw OR ("pain management"):ti,ab,kw OR ("pain assessment"):ti,ab,kw OR ("pain recognition"):ti,ab,kw  #3 #1 AND #2 |

**Table S4**. Summary of public pain databases.

|  | Subjects |  |  | Data |  |  |
| --- | --- | --- | --- | --- | --- | --- |
| Database | Participants | Pain Source | Recorded Total | Modalities | Pain Labelling | Year |
| UNBC McMaster Shoulder Pain Archive | 25^a^ | shoulder pain | 200 sequences, 48398 frames | facial videos: RGB | Frame:16 level PSPI; Sequence:11 level VAS, OPR, AFF, SEN | 2011 |
| BioVid Heat Pain Database Part A | 87 | thermal stimuli | 8700 videos | facial videos: RGB | Frame: 4 level thermal stimulus intensity | 2013 |
| MIntPain | 20 | electrical stimuli | 9366 videos, 187939 frames | facial videos: RGB-Depth-Thermal | Frame: 5 level electrical stimuli | 2017 |
| EmoPain | 50 | chronic lower back pain | 585487 frames | facial and body videos: RGB; EMG; Audio; Motion; | Frame: 0-1 continuous | 2016 |
| DISFA | 27 | None | 130000 frames | facial videos: RGB | Frame: AUs and intensities | 2013 |
| ^a^: the authors had 129 participants, yet the released database included video sequences from 25 subjects  PACSLAC-II: pain assessment checklist for limited ability to communicate-II | | | | | | |

**Table S5**. Reported items and explanations.

| Supplement table 5. Reported items and explanations | | |
| --- | --- | --- |
| item | | explanation |
| Author and Year | | First author and year of publication |
| model design | |  |
|  | Facial feature descriptor | How features were extracted from facial images, can be algorithms or deep models |
|  | Temporal features | A 3-class index distinguishing how temporal information was included. The 3 classes correspond to no temporal info, info extracted by comparing 2 separate images, and info extracted by convolution of a series of images |
|  | Feature transformation | How extracted features were processed before input to a classifier |
|  | Classification method | The architecture or the classifier, e.g., SVM, CNN. |
| model training and validation | |  |
|  | Database | The Database used for model training and validation |
|  | Objects | The Objects used for model training and validation, based on the database, can be images, video frames and video sequences. Images differ from video frames due to the recording process of database; images were taken as photos, frames were extracted from a video sequence |
|  | Output Levels | The number of discrete pain intensities of the model output |
|  | validation method | The validation method used, can be hold-out (split) and cross-validation |
|  | external validation | If external validation was performed |
|  | evaluation metrics | The evaluation metrics and results of validation |
| meta-analysis | |  |
|  | True Positive, TP | the number of images/frames that were correctly classified, the output intensity matches the defined threshold |
|  | False Positive, FP | the output intensity matches the threshold while the ground truth does not |
|  | False Negative, FN | the output intensity does not match the threshold while the ground truth does |
|  | True Negative, TN | the output intensity does no match the threshold and so does the ground truth |
