# Supplementary figures and images for: Application of AI in Multilevel Pain Assessment Using Facial Images: Systematic Review and Meta-Analysis
Source: J Med Internet Res. 2024 Apr 12;26:e51250. doi: 10.2196/51250 (PMC11053395; doi:10.2196/51250)

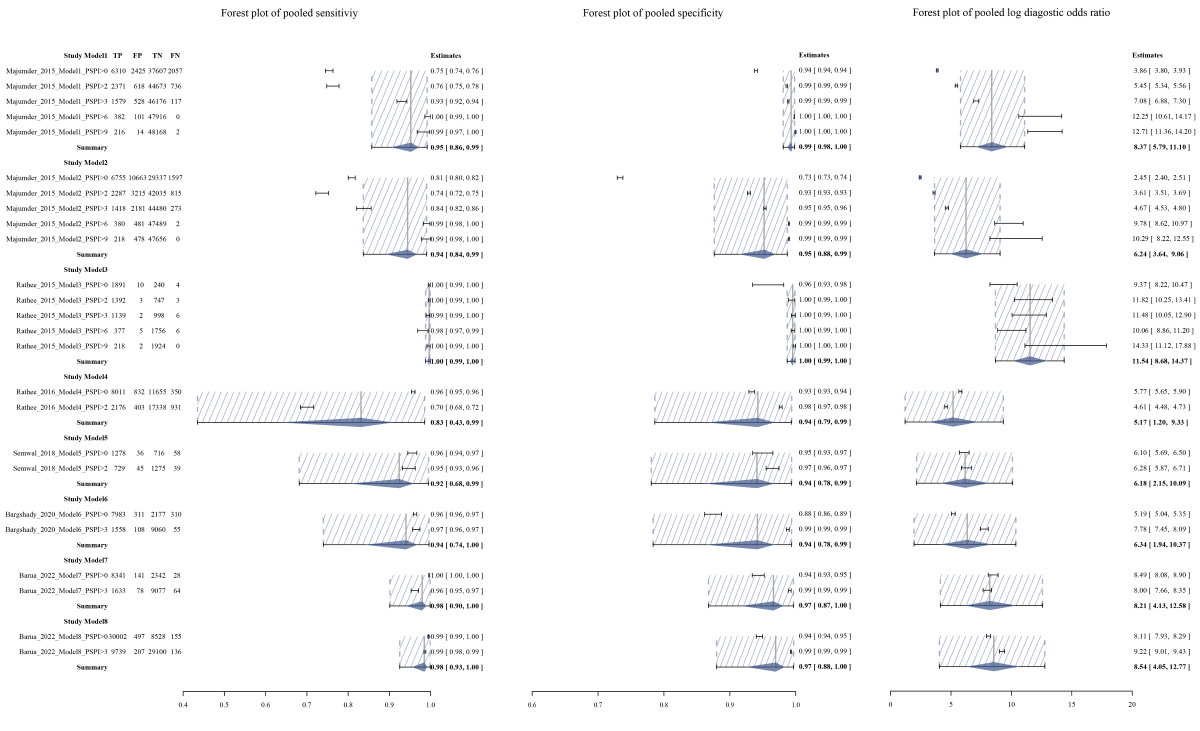

Supplement: Multimedia Appendix 3 [file jmir_v26i1e51250_app3.png]
